# Polysaccharides from Liriopes Radix ameliorate streptozotocin-induced type I diabetic nephropathy via regulating NF-κB and p38 MAPK signaling pathways

**DOI:** 10.1186/1472-6882-14-156

**Published:** 2014-05-13

**Authors:** Hung-Jen Lu, Thing-Fong Tzeng, Shorong-Shii Liou, Sheng Da Lin, Ming-Chang Wu, I-Min Liu

**Affiliations:** 1 Department of Food Science, College of Agriculture, National Pingtung University of Science and Technology, Neipu Township, Pingtung County, Taiwan; 2 Department of Pharmacy & Graduate Institute of Pharmaceutical Technology, Tajen University, Yanpu Township, Pingtung County, Taiwan

## Abstract

**Background:**

The polysaccharides from Liriopes Radix (PSLR) has been indicated to ameliorate insulin signaling transduction and glucose metabolism. We aimed to investigate whether PSLR exerts an ameliorative effect on renal damage in diabetes induced by streptozotocin.

**Methods:**

Diabetes was induced with STZ (60 mg/kg) by intraperitoneal injection in rats. Two weeks after STZ injection, rats in the treatment group were orally dosed with PSLR (200 and 300 mg/kg/day for 8 weeks. The normal rats were chosen as nondiabetic control group. Changes in renal function-related parameters in plasma and urine were analyzed at the end of the study. Kidneys were isolated for pathology histology, immunohistochemistry, and Western blot analyses.

**Results:**

Diabetic rats exhibited renal dysfunction, as evidenced by reduced creatinine clearance, blood urea nitrogen and proteinuria, along with marked elevation in the ratio of kidney weight to body weight. All of these abnormalities were significantly reversed by PSLR. The histological examinations revealed amelioration of diabetes-induced glomerular pathological changes following treatment with PSLR. The less protein expressions of renal nephrin and podocin in diabetic rats were increased following treatment with PSLR. PSLR reduced the accumulation of ED-1-expressing macrophages in renal tissue of diabetic rats. PSLR almost completely abolished T cells infiltration and attenuated the expression of proinflammatory cytokines. PSLR treatments not only reduced the degradation of inhibitory kappa B kinase, but also downregulated the protein expression of nuclear factor kappa B (NF-κB) and p38 mitogen-activated protein kinase (MAPK) in diabetic kidney.

**Conclusions:**

The results suggest that the renal protective effects of PSLR occur through improved glycemic control and renal structural changes, which are involved in the inhibition of NF-κB and p-38 MAPK mediated inflammation.

## Background

Diabetes mellitus is a metabolic disease usually characterized by the classic triad of polydipsia, polyuria and polyphagia, consequences of homeostasis disruption due to impaired glucose metabolism [1]. Diabetic nephropathy (DN), the second most prevalent diabetes-associated complication inferior to cardiovascular disorders, impaired the renal function of diabetes patients and therefore cost appreciable medical labor and resource for DN management annually [2]. Although DN is traditionally considered a non-immune disease, accumulating evidence now indicates that immunologic and inflammatory mechanisms play significant roles in its development and progression [3]. It also suggested the development of DN was associated with the activation of several signal pathways, including nuclear factor kappa B (NF-κB) and mitogen-activated protein kinase (MAPK) [4]. Genetic and pharmacological approaches that reduce inflammation in DN have not only enhanced our understanding of the disease pathophysiology, but have also shown promise as potential therapeutic strategies.

Many herbal extracts or derivatives have been documented in traditional Chinese medicine (TCM) as having clinical effectiveness in treating diabetes mellitus [5]. The tuberous root of *Liriope spicata* (Liliaceae) were recorded as Liriopes Radix in the Pharmacopeia of the People’s Republic of China, are frequently used as “maidong” in prescriptions for the treatment of diabetes mellitus. Chemical studies have shown that this plant mainly includes saponins, polysaccharide and homoisoflavonoidal compounds [6]. It has been reported that the water extract and crude polysaccharides obtained from Liriopes Radix had considerable hypoglycemic effects [7]. The polysaccharides from Liriopes Radix (PSLR) caused more marked activation on insulin signaling transduction in type 2 diabetic mice than the effects produced by water extract has been also demonstrated [8]. Liriopes Radix is valued for the ability to promote glucose homeostasis, and it may therefore be utilized as an adjuvant therapy in the control of diabetic complications. The potential preventive effects of PSLR on DN in diabetic rats has been reported to mediate by down-regulating the system of advanced glycation end products-receptor for advanced glycation end products [9]. Actually, anti-inflammatory potential of Liriopes Radix has been identified [10]. Since the inflammatory process is also involved in the pathogenesis of DN, there is a possibility that PSLR may ameliorate DN by suppression of renal inflammation, but this has never been explored.

Without intervention, approximately 80% of patients with type 1 diabetes and 20-40% of those with type 2 diabetes develop overt nephropathy in 10–15 years [11]. The present studies were thus designed to further elucidate whether the renopreventive effects of PSLR be responsible for the alleviation of diabetic renal inflammation utilizing streptozotocin-induced diabetic rats (STZ-diabetic rats) as a type-1 diabetic animal model.

## Methods

### Plant material and extraction

The tuberous root of *L. spicata* was purchased from a local market in Pingtung County (Taiwan) on May 2012. Plant identification was done by Professor Hong T.Y. (the Department of Biotechnology, Collage of Pharmacy and Health Care, Tajen University). Random amplified polymorphic DNA analysis of Liriopes Radix supplied was also performed to identify DNA polymorphisms. The voucher specimen (Lot No. LS20120523) has been deposited in our laboratory.

Preparation of PSLS was performed according to the previous study [7]. The powdered material (200 g) was boiled in distilled water three times (1:4, 1:4, 1:2, w/v), 0.5 h each time. Each extract was then filtered and merged together. Phosphate buffer (pH 5.91) and 0.6 g of papain (12 u/mg, Biosharp, USA) were added into the extracts and kept in water-bath (45°C) for 2 h to remove proteins. After boiled for 5 min, the extracts were stored overnight at 4°C and filtered next morning. The filtrate was purified by using the mini pellicon system (ultrafiltration membrane Mw cut-off 1000, Millipore, USA) with 12 l distilled water. The retentate portion was concentrated and then separated in DEAE-cellulose 52 columns to remove pigments. The water eluted solution was concentrated and finally lyophilized (Alpha 2–4, Christ, Germany) and obtained 53.2 g of PSLR from 200 g powdered material. The sugar content of PSLR was 98.1% determined by anthrone-sulfuric acid method at 625 nm using fructose as standard [12].

### Animal models

Male Wistar rats (8–10 weeks of age, 200–250 g) were obtained from the Animal Center of National Cheng Kung University Medical College. To induce diabetes rats were given a single intravenous injection of 60 mg/kg streptozotocin (STZ; Sigma-Aldrich, Inc., Saint Louis, Missouri, USA). Animals were considered to be diabetic if they had plasma glucose concentrations of 350 mg/dl or greater, in addition to polyuria and other diabetic features. All studies were carried out two weeks after the injection of STZ. All animal procedures were performed according to the Guidelines for the Care and Use of Laboratory Animals of the National Institutes of Health (United States), as well as the guidelines of the Animal Welfare Act. These studies were conducted with the approval of the Institutional Animal Care and Use Committee (IACUC) at Tajen University (approval number: IACUC 100–29; approval date: December 22, 2011).

### Treatment protocols

The selection of dosage regime for the present studies was according to the previous report that demonstrated administration with PSLR at 200 and 300 mg/kg for 4 weeks exerted potential effect in improving hyperglycemia in diabetic mice [9]. In order to obtain the significant effect of PSLR on improvement of DN, STZ-diabetic rats were dosed by oral gavage once per day for eight weeks with PSLR doses of 200 or 300 mg/kg in a volume of 1.5 ml/kg distilled water. A vehicle-treated group of STZ-diabetic rats and normal rats was treated with 1.5 ml/kg distilled water only over the same treatment period. Animals received standard rat diet (Harlan Teklad, Madison, WI, USA; Cat. No. 2018) and water ad libitum throughout the entire treatment period. PSLR treatment was continued even though the plasma glucose of STZ-diabetic rats was lower than 350 mg/dl during the eight-week treatment period. The evening prior to blood sample collection animals were restricted to 3 g of chow (given at 18:00), which was consumed immediately, and thereafter had access to only water. The animals were transferred to metabolic cages (Shineteh Instruments Co., Ltd, Taipei, Taiwan) for urine collection 24 hours before sacrifice. Urine was collected under a layer of toluene (to inhibit bacteria growth) and stored at 4°C until analyzed. Toluene used had no detectable effect on the estimation of albumin and creatinine in the urines collected in the metabolism experiments.

At the end of the eight-week treatment, rats were sacrificed using an intraperitoneal injection of sodium pentobarbital (50 mg/kg). The kidneys were dissected and rinsed with cold isotonic saline and weighed. An index of renal hypertrophy was estimated by comparing the wet weight of the left kidney to total body weight. The cortical tissues from right kidney were stored immediately at -80°C in liquid nitrogen for biochemical determinations and Western blot analyses. Other kidney tissues were fixed in 10% neutralized formalin for histology and immunohistochemistry.

### Blood sampling and analysis

Blood sample of rats were centrifuged at 2,000 × *g* for 10 minutes at 4°C, plasma was removed and aliquot for the respective analytical determinations. The diagnostic kit for determinations for plasma levels of glucose (Cat. No. COD12503) was purchased from BioSystem (Barcelona, Spain). The plasma creatinine (Cr) concentration was determined by the commercial assay kit (Cat. No. 221–30) purchased from Diagnostic Chemicals Limited (Connecticut, USA). Blood urea nitrogen (BUN) was determined by kinetic reagent (Diagnostic Chemicals Limited, Cat. No. 283–30). Commercial enzyme-linked immunosorbent assay (ELISA) kits were used quantify glycosylated hemoglobin (HbA_1c_) levels (Intergrated Bio Ltd., Taipei, Taiwan; Cat. No. CSB-E08140r). Kits for determination of plasma alanine aminotransferase (ALT; EC 2.6.1.2) (Cat. No. A524-780TM) and aspartate aminotransferase (AST; EC 2.6.1.1) (Cat. No. A559-780TM) concentrations were purchased from Teco Diagnostics (CA). Assays were performed according to the manufacturer’s instructions. All experiments were performed in triplicate to ensure the accuracy of the observations.

### Analysis of urine parameters

The 24-h urine collected from each diabetic rat and age-matched control was centrifuged at 2,000  *g* for 10 min. Urinary albumin concentrations were measured by Nephrat II ELISA kit (Cat. No. NR002) obtained from Exocell, INC. (PA, PUA). The concentration of Cr in pooled urine samples was determined by the commercial assay kit (Diagnostic Chemicals Limited, Cat. No. 221–30). All analyses were performed in triplicate in accordance with the manuals provided by the manufacturers. Creatinine clearance (Ccr) was calculated in individual rats as follows: Ccr = urine creatinine × urine volume/plasma creatinine × time [13].

### Renal cytokines determination

Renal tissue was homogenized in 10 mmol/L Tris–HCl buffered solution (pH 7.4) containing 2 mol/l NaCl, 1 mmol/l EDTA, 0.01% Tween 80, 1 mmol/l PMSF, and centrifuged at 9000 × *g* for 30 min at 4°C [14]. The resultant supernatant was used for cytokine determination. The levels of proinflammatory and anti-inflammatory cytokines were estimated by performing ELISA using commercial kits. ELISA kits for the determination of tumor necrosis factor (TNF)α (Cat. No. ab46070), interleukin (IL)-6 (Cat. No. ab100772), and IL-10 (Cat. No. ab100764) were obtained from Abcam Inc. (Cambridge, MA, USA). Samples were assayed in triplicate according to manufacturer’s instructions. The protein concentrations of kidney filtrate were determined according to the previous method using BSA as a standard [15].

### Renal histological analysis

Renal tissues were fixed with 10% neutral formalin phosphate buffer, dehydrated through a graded alcohol series and embedded in paraffin, cut into 4 μm sections and stained with hematoxylin and eosin (H&E). The sections were examined with light microscopy by an experienced pathologist and micrographs from six glomerulums were obtained randomly with magnification of 400 ×. Mean glomerular volume was determined from the mean glomerular capillary tuft area (A_G_) by light microscopy of PAS sections. The areas were determined by light microscopy and analyzed by dedicated software (Analysis 3.0, Soft Imaging System, Münster, Germany) as the average area of 50 glomerular profiles (the capillary tuft omitting the proximal tubular tissue and Bowman’s capsule) for each animal. Glomerular volume (GV) was calculated using the formula GV = β/k × (A_G_)^3/2^, where GV is glomerular volume, β = 1.38, which is the shape coefficient for spheres (the idealized shape of glomeruli), k = 1.1, which is a size distribution coefficient, and A_G_ is the glomerular capillary tuft area [16]. The index of mesangial expansion was scored by a quantitative estimate of the mesangial zones width in each glomerulus, expressed as a function of the total glomerular area [17]: 0, normal glomeruli; 1, matrix expansion occurred in up to 50% of a glomerulus; 2, matrix expansion occurred in 50-75% of a glomerulus; 3, matrix expansion occurred in 75-100% of a glomerulus.

### Immunohistochemistry

Formalin-fixed, paraffin-embedded kidney tissue sections were used for immunohistochemical staining. After deparaffinization and hydration, the slides were washed in Tris-buffered saline (TBS; 10 mmol/L Tris HCl, 0.85% NaCl, pH 7.2). Endogenous peroxidase activity was quenched by incubating with the slides in methanol and 0.3% H_2_O_2_ in methanol. After overnight incubation with Mouse monoclonal anti-rat monocyte/macrophage antibody (anti-ED-1) (sc-59103; Santa Cruz Biotechnology Inc. CA, USA) at 4°C, the slides were washed in TBS and horseradish peroxidase (HRP)-conjugated goat anti-mouse secondary antibody was then added and the slides were further incubated at room temperature for 1 h. The slides were washed in TBS and incubated with diaminobenzidine tetrahydrochloride as the substrate, and counterstained with hematoxylin. A negative control without primary antibody was included in the experiment to verify the antibody specificity. Intraglomerular ED-1-positive cells were counted in 200 glomeruli/group under 400-fold magnification.

### Western blotting

Protein extraction of isolated kidney was performed as follows [18]. The sample was homogenized in ice-cold in 1 ml of hypotonic buffer A [10 mmol/l HEPES (pH 7.8), 10 mmol/l KCl, 2 mmol/l MgCl_2_, 1 mmol/l DTT, 0.1 mmol/l EDTA, 0.1 mmol/l phenylmethylsulfonylfluoride]. A solution of 80 μl of 10% Nonidet P-40 was added to the homogenates, and the mixture was centrifuged for 2 min at 14,000 × *g*. The supernatant was collected as a cytosolic fraction for the assays of CD4, CD8, nephrin, podocin, and inhibitory kappa B kinase (IκB)α. The supernatant containing nuclear proteins was collected for nuclear factor kappa B (NF-κB) p65, p38 mitogen-activated protein kinase (p38 MAPK), and phospho (p)-p38 MAPK (Thr180/Tyr182).

Before immunoblotting, and the protein concentration of each tissue was determined using a Bio-Rad protein assay kit (Bio-Rad Laboratories, Japan) and bovine serum albumin as a standard, to ensure equal loading among lanes. Cytosol (70 μg total protein) and nuclear extracts (50 μg total protein) were separated on a 7.5-15% polyacrilamide gel and electophoretically transferred to nitrocellulose membrane. Membranes were blocked with 5% non-fat dry milk in Tris-buffered saline Tween (20 mmol/l Tris, pH 7.6, 137 mmol/l NaCl, and 0.1% Tween 20) for 3 h at room temperature, followed by an overnight incubation at 4°C with with primary antibodies CD4 (Santa Cruz Biotechnology, Inc., Cat. No. sc-7219), CD8 (Santa Cruz Biotechnology, Inc., Cat. No. sc-7188), nephrin (Santa Cruz Biotechnology, Inc., Cat. No. sc-28192), podocin (Santa Cruz Biotechnology, Inc., Cat. No. sc-21009), NF-κB p65 (Santa Cruz Biotechnology, Inc., Cat. No. sc-109), IκBα (Cell Signaling Technology, Beverly, MA, USA; Cat. No. 9242), p38 MAPK (Cell Signaling Technology; Cat. No. 9212), p-p38 MAPK (Thr180/Tyr182) (Cell Signaling Technology; Cat. No. 9211), or β-actin (Santa Cruz Biotechnology, Inc.; Cat. No. sc-130656). All antibodies were used at a dilution of 1:1000. Three times after washing with Tris-buffered saline Tween 20 (TBST), incubation with appropriate horseradish peroxidase-conjugated secondary antibodies were performed for 1 h at room temperature. After three additional TBST washes, the immunoreactive bands were visualized by enhanced chemiluminescence (Amersham Biosciences, Buckinghamshire, UK) according to the manufacturer’s instructions. Band densities were determined using ATTO Densitograph Software (ATTO Corporation, Tokyo, Japan) and quantified as the ratio to β-actin. The mean value for samples from the vehicle-treated normal rats on each immunoblot, expressed in densitometry units, was adjusted to a value of 1.0. All experimental sample values were then expressed relative to this adjusted mean value. Tissue sections were sampled from 4 independent experiments.

### Statistical analysis

The results are presented as the mean ± standard deviation (SD) for each group of animals at the number (*n*) indicated. Statistical analysis was performed with one-way analysis of variance (ANOVA). The Dunnett range post-hoc comparisons were used to determine the source of significant differences where appropriate. The renal morphohistology and the morphologic analysis for PAS staining were analyzed statistically using the Kruskal-Wallis Test and Dunn’s Multiple Comparisons Test. Values of P < 0.05 were considered statistically significant.

## Results

### Effects on body weight, fasting blood glucose and glycosylated hemoglobin contents

During 8-week experiment, STZ-diabetic rats were found to have significant weight loss when compared with normal rats (Table 1). The body weight reduction was not obvious in STZ-diabetic rats receiving PSLR during the experimental period (Table 1).

**Table 1:** Biochemical parameters and the renal levels of cytokines in experimental animals at the end of the eight-week treatment

	**Normal rats**	**STZ-diabetic rats**		
	**Vehicle**	**Vehicle**	**PSLR 200**	**PSLR 300**
Body weight (BW) (g/rat)	370.39 ± 13.58^d^	240.99 ± 16.24^b^	264.25 ± 15.11^b,c^	307.72 ± 14.72^a,c^
Kidney weight (KW) (g)	1.29 ± 0.23^c^	2.43 ± 0.17^a^	1.85 ± 0.24^a^	1.52 ± 0.16
KW/BW ratio × 1000	3.48 ± 0.19^d^	10.08 ± 0.17^b^	7.00 ± 0.12^b,c^	4.94 ± 0.11^b,c^
Plasma glucose (mg/dl)	92.68 ± 8.59^d^	425.73 ± 18.07^b^	336.25 ± 14.92^b,c^	306.92 ± 15.31^b,c^
HbAlc (%)	4.79 ± 1.03^d^	14.31 ± 1.26^b^	11.75 ± 1.18^b^	10.91 ± 1.21^b^
Urine volume (ml/day)	9.26 ± 2.43^d^	28.16 ± 9.13 ^b^	18.21 ± 6.28^b,c^	15.84 ± 7.31^b,c^
Urine protein (mg/day)	6.95 ± 2.84^d^	34.83 ± 5.29^b^	16.41 ± 4.13^a,c^	12.27 ± 5.21^d^
Plasma Cr (μmol/l)	38.50 ± 6.34^d^	96.29 ± 8.73^b^	84.10 ± 8.31^b^	67.92 ± 7.32^b,c^
BUN (mmol/l)	7.21 ± 1.58^c^	16.29 ± 2.80^a^	13.16 ± 2.11^a^	11.45 ± 2.21^a,c^
Ccr (ml/min)	3.88 ± 0.89^d^	1.65 ± 0.54^b^	2.37 ± 0.68^a,c^	2.77 ± 0.34^d^
Plasma AST (U/l)	125.85 ± 10.47^d^	374.68 ± 17.34^b^	219.88 ± 18.26^b,c^	159.57 ± 17.53^d^
Plasma ALT (U/l)	57.67 ± 7.92^d^	218.67 ± 17.12^b^	145.14 ± 15.78^b,c^	103.42 ± 13.24^a,d^
Renal IL-6 (pg/mg protein)	54.06 ± 8.46^d^	159.34 ± 11.38^b^	126.34 ± 10.28^b,c^	105.89 ± 10.94^b,c^
Renal TNF-α (pg/mg protein)	69.19 ± 9.34^d^	172.91 ± 10.11^b^	137.12 ± 9.13^b,c^	109.45 ± 7.51^b,c^
Renal IL-10 (pg/mg protein)	64.77 ± 4.17^d^	28.43 ± 4.25^b^	36.49 ± 3.91^b,c^	44.64 ± 3.77^b,c^

STZ-diabetic rats were dosed by oral gavage once per day for eight weeks with 200 mg/kg/day PSLR (PSLR 200), 300 mg/kg/day PSLR (PSLR 300). Normal or STZ-diabetic rats receiving vehicle treatment were given the same volume of vehicle (distilled water) used to disperse PSLR. Values (mean ± SD) were obtained for each group of 8 animals. ^a^P < 0.05 and ^b^P < 0.01 compared to the values of vehicle-treated normal rats, respectively. ^c^P < 0.05 and ^d^P < 0.01 compared to the values of vehicle-treated STZ-diabetic rats, respectively.

Significant increase in fasting blood glucose in STZ-diabetic was observed when compared to normal control group and this change was more marked at the 8th week following diabetes induction. The blood glucose lowering effect was obvious when STZ-diabetic rats were treated with PSLR at the daily dosage of 200 mg/kg (20.91 ± 4.12%) and 300 mg/kg (28.37 ± 3.92%) for 8 weeks (Table 1). The value of HbA_1c_ was markedly higher in STZ-diabetic rats (14.31 ± 1.26%) when compared with theat from normal rats (4.79 ± 1.03%, Table 1). Treatment PSLR at the daily dosage of 200 and 300 mg/kg for 8 weeks decreased the levels of HbA_1c_ in STZ-diabetic rats by 17.88 ± 3.29% and 23.75 ± 2.86% relative to the value in STZ-diabetic rats that received vehicle, respectively (Table 1).

### Effects on renal and hepatic function related parameters

STZ-diabetic rats showed an increase in 24 h urine volume, accompanied by increase in urine protein excretion (Table 1). After 8 weeks of 300 mg/kg/day PSLR treatment, 24 h urine volume and 24 h urine protein excretion of STZ-diabetic rats were markedly less than their vehicle-treated counterparts (Table 1). The levels of Scr and BUN in STZ-diabetic rats were obviously higher than normal control group. There was an effective reduction in the levels of Scr and BUN in STZ-diabetic rats receiving 8 weeks of PSLR treatment when compared with their vehicle-counterparts (Table 1). In addition, increased Ccr in STZ-diabetic rats was observed after 8 weeks of PSLR treatment (Table 1).

Plasma ALT and AST activities in the diabetic control group were significantly higher than those in the normal control group (Table 1). The ALT and AST activities were markedly reduced in STZ-diabetic rats treated for eight weeks with PSLR (Table 1).

### Influences on the kidney hypertrophy

The mean weight of the left kidney and the ratio of kidney weight to body weight in vehicle-treated STZ-diabetic rats were increased significantly compared with those in the normal group. Treatment of STZ-diabetic rats with 200 mg/kg/day PSLR slightly reduced the degree of renal hypertrophy (Table 1). Kidney hypertrophy and the ratio of kidney weight to body weight were markedly reduced in STZ-diabetic rats treated for eight weeks with 300 mg/kg/day PSLR (Table 1).

### Influences on the renal cytokines

Renal concentrations of inflammatory cytokines TNF-α and IL-6 of STZ-diabetic rats were significantly higher compared to their vehicle-treated controls (Table 1). PSLR treatments reduced renal IL-6 and TNF-α levels in a dose-dependent manner (Table 1).

The renal IL-10 level of STZ-diabetic rats was significantly lower than that in the normal control (Table 1). The decreased renal IL-10 level in the kidneys of STZ-diabetic rats was enhanced dose-dependently by PSLR treatment (Table 1).

### Influences on the renal histology

**Figure 1:**
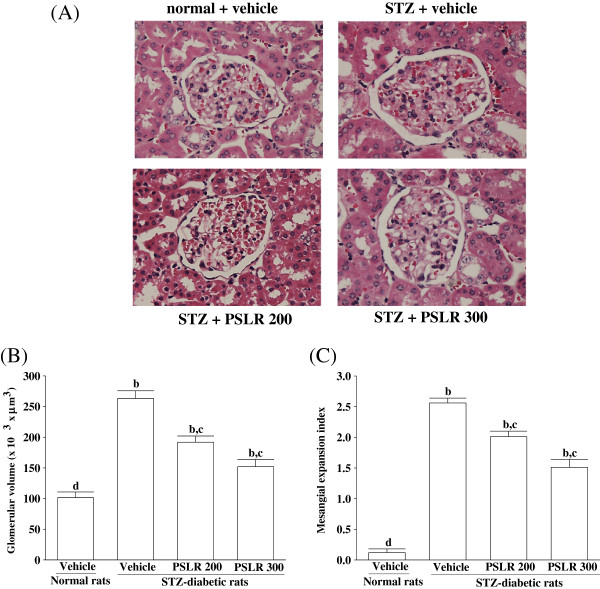
**Effects of treatments on the renal histology. (A)** Representative photomicrographs (original magnification, 400×) of H&E-stained kidney sections, **(B)** glomerular volume, and **(C)** expansion of matrix index expressed as a quantitative estimate score in experimental groups. STZ-diabetic rats were dosed by oral gavage once per day for eight weeks with 200 mg/kg PSLR (STZ + PSLR 200) or 300 mg/kg PSLR (STZ + PSLR 300). Normal or STZ-diabetic rats receiving vehicle treatment were given the same volume of vehicle (distilled water) used to to disperse PSLR. Values (mean ± SD) were obtained for each group of 4 animals. ^b^P < 0.01 compared to the values of vehicle-treated normal rats (normal + vehicle). ^c^P < 0.05 and ^d^P < 0.01 compared to the values of vehicle-treated STZ-diabetic rats (STZ + vehicle), respectively.

The changes in renal histology of the different groups are shown in Figure 1A. The sections from the normal control group were normal sizes without any abnormal phenomena by visual observation (Figure 1A). Conversely, glomerular proliferation and mesangial matrix augmentation occurred in the diabetic group. These broad changes caused loading of the Bowman’s capsule space and adhesion of capillaries to the wall (Figure 1A). The structures of the kidney sections of PSLR (300 mg/kg/day)-treated groups also changed in morphology, however, a decreased extent of the expansions in the glomerulus and the mesangial matrix were observed (Figure 1A).

When a semiquantitative analysis was used, it became readily apparent that a difference in the histological change was present the diabetic control group and other groups. The glomerular volume and the extent of mesangial matrix nearly increased to 2.61- and 2.56-fold in diabetic control group compared with the normal control group, respectively (Figure 1B and C). The glomerular volume and the extent of mesangial matrix in PSLR (300 mg/kg/day)-treated STZ-diabetic rats were decreased 42.58 ± 5.62 and 41.01 ± 6.25%, respectively, relative to those in diabetic control group (Figure 1B and C).

### Effects on macrophage infiltration

**Figure 2:**
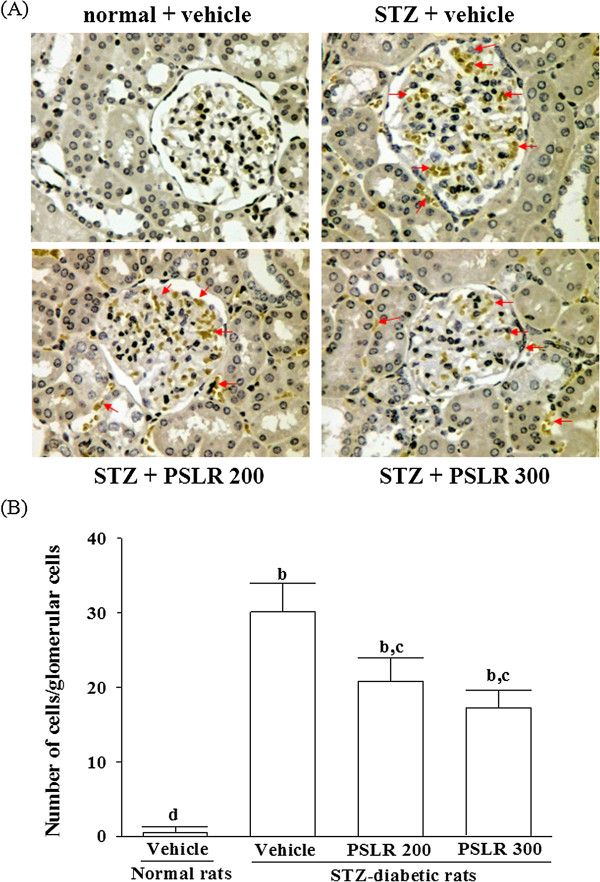
**Effects of treatments on macrophage infiltration. (A)** Immunohistochemistry staining for macrophage (ED-1-positive cells) in the renal tissues of STZ-diabetic rats. STZ-diabetic rats were dosed by oral gavage once per day for eight weeks with 200 mg/kg PSLR (STZ + PSLR 200) or 300 mg/kg PSLR (STZ + PSLR 300). Normal or STZ-diabetic rats receiving vehicle treatment were given the same volume of vehicle (distilled water) used to to disperse PSLR. Arrows indicate ED-1-positive cells. **(B)** Quantification results are shown for number of macrophages (ED-1-positive cells). Values (mean ± SD) were obtained for each group of 4 animals. ^b^P < 0.01 compared to the values of vehicle-treated normal rats (normal + vehicle). ^c^P < 0.05 and ^d^P < 0.01 compared to the values of vehicle-treated STZ-diabetic rats (STZ + vehicle), respectively.

Kidneys from control rats did not show any significant macrophage infiltration; however, STZ-diabetic rats demonstrated prominent macrophage (ED-1-positive cells) infiltration in the glomerulus (Figure 2). Treatment of STZ-diabetic rats with 300 mg/kg/day PSLR for eight weeks caused a 42.76 ± 6.22% reduction of macrophage influx relative to that in their vehicle-treated counterparts (Figure 2).

### Effects on renal T cells expression

**Figure 3:**
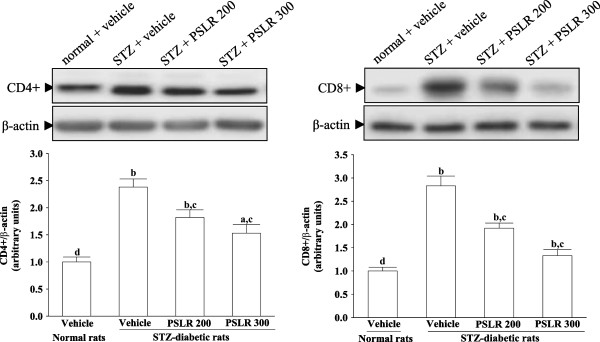
**Effects of treatments on protein expressions of CD4+ and CD8+ T cells in the renal tissues of STZ-diabetic rats.** STZ-diabetic rats were dosed by oral gavage once per day for eight weeks with 200 mg/kg PSLR (STZ + PSLR 200) or 300 mg/kg PSLR (STZ + PSLR 300). Normal or STZ-diabetic rats receiving vehicle treatment were given the same volume of vehicle (distilled water) used to to disperse PSLR. Values (mean ± SD) were obtained for each group of 4 animals. ^a^P < 0.05 and ^b^P < 0.01 compared to the values of vehicle-treated normal rats (normal + vehicle), respectively. ^c^P < 0.05 and ^d^P < 0.01 compared to the values of vehicle-treated STZ-diabetic rats (STZ + vehicle), respectively.

Renal CD4+ cells protein expression was 3.1-fold higher in STZ-diabetic rats compared with normal control, and 300 mg/kg/day PSLR treatment attenuated this increase by 64.28 ± 5.71% (Figure 3A). The STZ-induced upregulation of renal CD8 + T cells was reduced 53.01 ± 5.34% relative to that in vehicle-treated STZ-diabetic rats after eight weeks of treatment with 300 mg/kg/day PSLR (Figure 3B).

### Changes in protein expressions of nephrin and podocin

**Figure 4:**
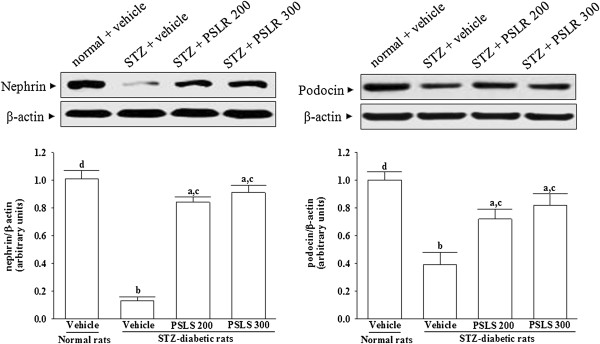
**Effects of treatments on protein expressions of nephrin and podocin in the renal tissues of STZ-diabetic rats.** STZ-diabetic rats were dosed by oral gavage once per day for eight weeks with 200 mg/kg PSLR (STZ + PSLR 200) or 300 mg/kg PSLR (STZ + PSLR 300). Normal or STZ-diabetic rats receiving vehicle treatment were given the same volume of vehicle (distilled water) used to to disperse PSLR. Values (mean ± SD) were obtained for each group of 4 animals. ^a^P < 0.05 and ^b^P < 0.01 compared to the values of vehicle-treated normal rats (normal + vehicle), respectively. ^c^P < 0.05 and ^d^P < 0.01 compared to the values of vehicle-treated STZ-diabetic rats (STZ + vehicle), respectively.

Western blot assay indicated that the renal nephrin and podocin proteins were less expressed in vehicle-treated STZ-diabetic rats (Figure 4). Eight weeks of PSLR (300 mg/kg/day) treatment resulted in a marked increase of renal nephrin and podocin protein expression in STZ-diabetic rats (Figure 4).

### Influences on the renal expression of NF-κB and IκBα

**Figure 5:**
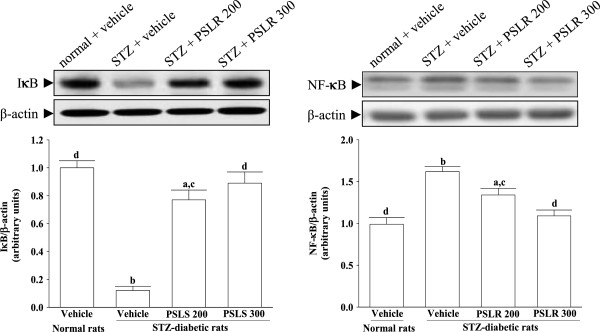
**Effects of treatments on protein expressions of renal IκB and NF-κB in the renal tissues of STZ-diabetic rats.** STZ-diabetic rats were dosed by oral gavage once per day for eight weeks with 200 mg/kg PSLR (STZ + PSLR 200) or 300 mg/kg PSLR (STZ + PSLR 300). Normal or STZ-diabetic rats receiving vehicle treatment were given the same volume of vehicle (distilled water) used to to disperse PSLR. Values (mean ± SD) were obtained for each group of 4 animals. ^a^P < 0.05 and ^b^P < 0.01 compared to the values of vehicle-treated normal rats (normal + vehicle), respectively. ^c^P < 0.05 and ^d^P < 0.01 compared to the values of vehicle-treated STZ-diabetic rats (STZ + vehicle), respectively.

As shown in Figure 5A, the cytosolic IκBα in the kidney of STZ-diabetic rats was lower than that in the normal group, and 300 mg/kg/day PSLR treatment reduced IκBα degradation. The accumulation of NF-κB proteins in the nucleus of glomeruli from vehicle-treated STZ-diabetic rats relative to normal group were observed in Figure 5B. The increased expression of NF-κB protein found in the nucleus of kidneys from STZ-diabetic rats was reduced after 8-week of 300 mg/kg/day PSLR treatment (Figure 5B).

### Effects on the protein expression and phosphorylation of p38 MAPK

**Figure 6:**
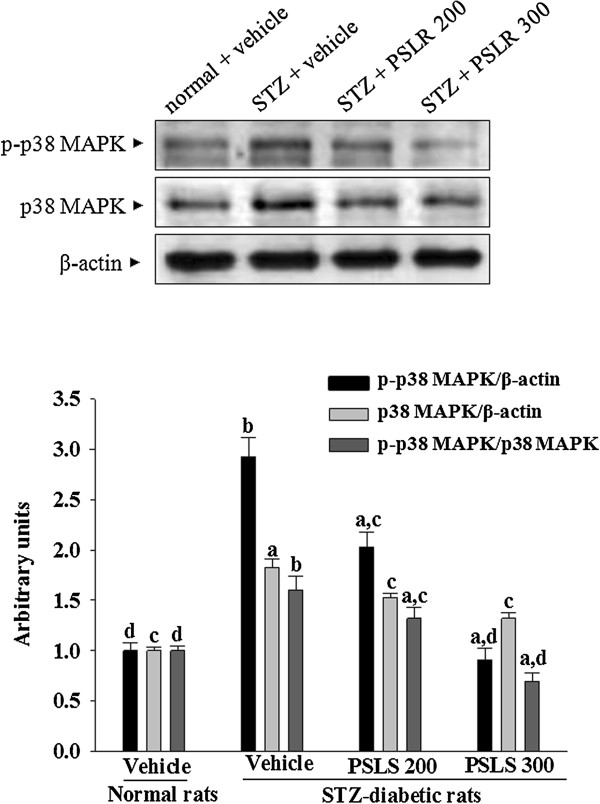
**Effects of treatments on protein expression and phosphorylation of p-38 MAPK in the renal tissues of STZ-diabetic rats.** STZ-diabetic rats were dosed by oral gavage once per day for eight weeks with 200 mg/kg PSLR (STZ + PSLR 200) or 300 mg/kg PSLR (STZ + PSLR 300). Normal or STZ-diabetic rats receiving vehicle treatment were given the same volume of vehicle (distilled water) used to to disperse PSLR. Ratios of p-p38 MAPK/β-actin, p38 MAPK/β-actin, or p-p38 MAPK/p38 MAPK are expressed as the mean with SD (n = 4 per group) in each column. ^a^P < 0.05 and ^b^P < 0.01 compared to the values of vehicle-treated normal rats (normal + vehicle), respectively. ^c^P < 0.05 and ^d^P < 0.01 compared to the values of vehicle-treated STZ-diabetic rats (STZ + vehicle), respectively.

The immunoblot results showed that the protein levels and phosphorylation degree of p38 MAPK were 1.81 and 2.93 fold higher in kidney of STZ-diabetic rats as compared to the normal group (Figure 6). The STZ also significantly increased the p-p38 MAPK/p38 MAPK ratio (by 1.62 fold relative to those in vehicle-treated normal rats) in kidney of the rats (Figure 6). These STZ-induced up-regulations in protein levels and phosphorylation degree of p38 MAPK were reversed in the kidney after 8-week treatment with 300 mg/kg/day PSLR by 31.05 ± 5.81 and 27.86 ± 6.24% decreases relative to those in vehicle-treated STZ-diabetic rats (Figure 6). The ratio of p-p38 MAPK/p38 MAPK were reversed in the kidney after 8-week treatment with 300 mg/kg/day PSLR by 43.12 ± 3.25% decreases relative to those in vehicle-treated STZ-diabetic rats (Figure 6).

## Discussion

In the present study, untreated STZ-diabetic rats developed severe hyperglycemia with polyuria as a result of osmotic diuresis. It is widely known that hyperglycemia can induce microalbuminuria by a hyperfiltration mechanism [19]. We found that the 8 week PSLR (300 mg/kg/day) administration regimen significantly inhibited the glycosylation of hemoglobin by lowering hyperglycemia in STZ-diabetic rats. The STZ-diabetic rats treated with PSLR showed an impressive decrease in the amount of proteinuria in parallel with the decrease in urinary volume, probably as a result of the amelioration of hyperglycemia as shown in other studies [7,8].

As the final and major size barrier to the passage of proteins and other macromolecules, slit diaphragm, the key structure of foot process, plays a crucial role in the occurrence and development of proteinuria [20]. Nephrin, a transmembrane protein belonging to the immunoglobulin superfamily of cell adhesion molecules, was the first identified structural protein of the podocyte slit diaphragm which has dramatic functional importance [21]. Attenuation of nephrin expression in experimental kidney diseases is associated with a loss of the slit diaphragm and massive proteinuria [21]. Podocin, a stomatin family member, is another important component of the glomerular slit diaphragm complex which colocalizes and interacts with cytosolic tail of nephrin in the lipid rafts of the podocyte foot process cell membrane [22]. Mutations in the podocin gene cause severe structural podocyte alterations and massive proteinuria leading to nephrotic syndrome [22]. It has been demonstrated that reduction of podocin leads to decreased expression and obvious redistribution of nephrin [23]. We thus examined whether antiproteinuric effects of PSLR correlated with alterations in the abundance of nephrin and podocin. Our observations demonstrated that rats with diabetes lost their functional podocin, exhibited less nephrin protein expressions, and developed proteinuria. This down regulated expression of renal nephrin and podocin protein was alleviated in the 8 weeks treatment with PSLR. In addition, PSLR treatment ameliorated proteinuria and glomerular pathological changes in diabetic kidney. We concededly demonstrated that the renal protective effect of PSLR might be related with the upregulation on nephrin and podocin expressions in the glomeruli from diabetic rats.

It is known that among inflammatory cytokines, such as TNF-α and IL-6 are relevant to the development of diabetic nephropathy, with diverse actions potentially involved in the development of complications [24]. Thus, the suppression on IL-6 and TNF-α could retard or alleviate inflammation and improve nephropathy. Our study found that the dietary intake PSLR could effectively decrease renal IL-6 and TNF-α levels in diabetic rats. These results similar with the effects of metformin to protect against DN by modulation of pro-inflammatory gene expression levels [25]. Antiinflammatory and immunosuppressive cytokines such as IL-10, could downregulate the production of proinflammatory cytokines from monocytes [26]. The elevation of IL-10 from PSLR supplement as observed in our present study might contribute to alleviate renal inflammatory stress. Therefore, our present study suggested that PSLR could alleviate diabetic renal inflammation via suppressing the release of IL-6 and TNF-α release, and increasing renal IL-10 levels.

T lymphocytes are known to interact with macrophages and regulate the inflammatory cascade [27]. Increased infiltration of monocytes/macrophages and activated T lymphocytes, as well as augmented expression of inflammatory cytokines in the kidneys have been found in patients with DN [28]. Using accumulation of ED-1 as a marker of macrophage activation, we have demonstrated increased activation of macrophage in the glomeruli of kidney tissue from diabetic animals. The suppressive effect from PSLR on the expression of ED-1 was observed. Western blot analyses revealed increased expression of CD4+, CD8 + T cells in diabetic kidneys were also allivated by PSLR supplement. Therefore, the possible mechanism of preventing the renal disease progression may be due to the effect of PSLR on attenuating inflammation through reducing macrophage and T cells infiltration in DN.

Studies have also demonstrated that NF-κB was involved in the induction of monocyte chemotactic protein-1 in mesangial cell cultured under high glucose condition and subsequently mediated macrophage accumulation [29]. NF-κB is present in the cytoplasma complexed to its inhibitory protein known as IκB. After activation by a number of physiological and nonphysiological stimuli, IκB dissociates from NF-κB within minutes and undergoes ubiquitination and degradation. Once NF-κB is released from the inhibitory unit IκB, the NF-κB is then translocated into the nucleus. Upon its nuclear translocation, NF-κB undergoes phosphorylation on serine 276 in its p65 subunit and associates with surrounding chromatin components. It subsequently binds with DNA and promotes the transcription of proinflammatory cytokines [30]. After PSLR treatment for 8 weeks, the nucleus protein levels of NF-κB p65 were significantly inhibited, implying the effect of PSLR on diabetic rats with renal injury may result from the inhibition of NF-κB activation. And in diabetic kidney, the cotosolic protein levels of IκBα were decreased, while PSLR treatment could up-regulated IκBα level and decreased the expression of inflammatory cytokines regulated by NF-κB, such as TGF-β1, TNF-α and IL-6, which increased in diabetic rats. Treated rats exhibited reduced levels of glucose and HbAc1. It should be noted that the significant influence of PSLR on hyperglycemia in STZ-diabetic rats was observed, supporting the traditional use of Liriopes Radix as a hypoglycemic agent [7,8]. We believe that the ability of PSLR to inactivate hyperglycemia-induced activation of NF-κB and thus inhibited the macrophages infiltration is its likely another mechanisms of action.

Accumulating evidence also suggested the development of DN was associated with the activation of several stress-sensitive signal pathways, including MAPK cascades. p38 MAPK followed by the activation of NF-κB participates in the intracellular signal transduction and production of cytokines and chemokines [31]. p38 MAPK was activated in vivo in glomeruli from diabetic rats and in vitro in mesangial cells exposed to high glucose [32]. Increased renal cortical p38 MAPK activity in diabetic rats could be attenuated by improved glycemic control [33]. Treatment with PSLR also reduced the elevated levels of p38 MAPK in the kidneys of STZ-diabetic rats, suggesting that the renal protective effect of PSLR might be also related with the modulation of p38 MAPK signal transduction to attenuate diabetes-affected renal dysfunction. We thus concluded that attainment of good glycemic control by PSLR treatment could abrogate the increased renal p38 MAPK pathway activation in diabetic rats and led to minimize risk of DN. This finding may support the protective role of p38 MAPK and NF-κB signaling pathways inhibition in the reduction of the development of DN. Taken together, all the above results suggest that the beneficial effect of PSLR in rats with DN is at least in part through antihyperglycemia which was accompanied by inhibition of macrophage infiltration via reducing NF-κB and p38 MAPK mediated inflammatory response. However, further pharmacological evaluations are required to isolate and identify the active principles in the plant as well as elucidating their mechanisms of action.

Toxicity studies on many herbal preparations have not been conducted. Although it is generally assumed that herbal preparations have fewer side effects than modern medicines; care should be taken with the chronic consumption of large doses of traditional remedies. ALT and AST are two of the most reliable markers of hepatocellular injury or necrosis [34]. We found that rats treated with PSLR exhibited no evidence of hepatotoxicity. Thus, PSLR could be considered with a margin of safety for oral use. Since toxicity in humans cannot always be entirely extrapolated from animal studies, clinical evaluation should be performed to precisely define the safe dosage to advice in humans.

## Conclusions

These results demonstrate that the renal protective effects of PSLR occur through improved glycemic control and renal structural changes, which are involved in the inhibition of NF-κB and p38 MAPK mediated inflammation. Given these promising preclinical findings, we believe that PSLR might be considered as potential adjuvant entity for DN treatment.

## Competing interests

The authors declare that they have no competing interests.

## Authors’ contributions

HJL carried out the experimentation as part of PhD study. MCW contributed to study design, data interpretation and manuscript writing. TTF and SDL performed the experiments and analysis and participated to data interpretation. SLL supervised the work and evaluated the data. IML supervised the work, evaluated the data, manuscript writing and corrected the manuscript for publication. All authors read and approved the final manuscript.

## Authors’ information

Ming-Chang Wu is the co-correspondence.

## Pre-publication history

The pre-publication history for this paper can be accessed here:

http://www.biomedcentral.com/1472-6882/14/156/prepub
